# Effects of Exercise on the Structure and Circulation of Choroid in Normal Eyes

**DOI:** 10.1371/journal.pone.0168336

**Published:** 2016-12-14

**Authors:** Takamasa Kinoshita, Junya Mori, Natsuki Okuda, Hiroko Imaizumi, Masanori Iwasaki, Miho Shimizu, Hirotomo Miyamoto, Kei Akaiwa, Kentaro Semba, Shozo Sonoda, Taiji Sakamoto, Yoshinori Mitamura

**Affiliations:** 1 Department of Ophthalmology, Institute of Biomedical Sciences, Tokushima University Graduate School, Tokushima, Japan; 2 Department of Ophthalmology, Sapporo City General Hospital, Sapporo, Japan; 3 Department of Ophthalmology, Kagoshima University Graduate School of Medical and Dental Sciences, Kagoshima, Japan; Universita degli Studi di Firenze, ITALY

## Abstract

**Aims:**

To determine the effects of dynamic exercise on the circulation and the luminal and stromal areas of the choroid in normal eyes.

**Methods:**

This was a prospective interventional study of 38 eyes of 38 normal subjects enrolled by invitation. The systolic and diastolic blood pressures, heart rate, intraocularpressure, mean ocular perfusion pressure (MOPP), choroidal blood velocity, and enhanced depth imaging optical coherence tomographic (EDI-OCT) images were recorded before, and immediately after mild dynamic exercise. The same measurements were recorded after 10 min of rest. The choroidal blood velocity was measured bylaser speckle flowgraphy, and the mean blur rate was used for the evaluations. The horizontal EDI-OCT images of the subfoveal choroid were converted to binary images. The central choroidal thickness (CCT), total cross sectional choroidal area, luminal areas, stromal areas, and the ratio of luminal area to total choroidal area (L/C ratio) were determined from these images.

**Results:**

The systolic and diastolic blood pressures, heart rate, MOPP, and the mean blur rate were significantly increased immediately after the exercise and significantly decreased 10 minutes after the exercise. There wereno significant changes in the mean CCT, the mean total choroidal area, the mean luminal and stromal areas, and the mean L/C ratio after the exercise.

**Conclusions:**

Our results suggest that a rest period is needed before measurements of blood flow velocity but not necessary for the EDI-OCT imaging to determine the choroidal thickness and area.

## Introduction

Exercise has become of great interest to many researchersbecause of its role in determining the quality of life. The effects of exercise on the systemic and ocular parameters have been studied extensively, and the ocular parameters studied included the intraocular pressure (IOP), ocular perfusion pressure, axial length, corneal thickness, anterior chamber depth, lens thickness, and blood flow in the ophthalmic artery, central retinal artery, optic nerve head, retina, and choroid [1–11].

The effects of exercise on the choroidal thickness are still controversial. The results of a recent study using the enhanced-depth imaging optical coherence tomography (EDI-OCT) showed that there was a significant increase in the choroidal thickness after exercise [12], but others did not find any significant changes in the thickness after the exercise [2, 13]. Because the choroid does not have a distinct layer-by-layer architecture, the changes of either the luminal or stromal areas of the choroid after exercise have not been determined.

Recently, a new method, called the binarization method, that can differentiate and quantify both the choroidal luminal and stromal areas has beendeveloped [14]. This method uses an open access software named ImageJ with a detailed protocol. The binarization technique has been used to differentiate the choroidal luminal area from the stromal area, and several studies have shown that not only the choroidal thickness but also the ratio of luminal area to total choroidal area (L/C ratio) changedwith ageing, diurnally, and differentially before and after the treatment in eyes with different ocular diseases [14–21].

To the best of our knowledge, there has not been a study that determined the changes in the luminal and stromal area of the choroid after exercise. This information should be useful in understanding the physiological changes in the choroid after exercise. Furthermore, it should be more informative when the structural changes in the choroid are evaluated in combination with the examination of choroidal blood flow.

Thus, the purpose of this study was to determine whether exercise will alter the luminal and stromal areas of the choroid. To accomplish this, we recorded EDI-OCT images before, immediately after dynamic exercise, and after 10 min of rest. The binarization technique was used to measure the luminal and stromal areas. Laser speckle flowgraphy (LSFG) was used to measure the choroidal blood flow velocity at the selected times.

## Material and Methods

Thisstudy conformed to the tenets of the Declaration of Helsinki, and a written informed consent was obtained from all of the subjects. Thestudy was approved by the Institutional Review Board of Sapporo City General Hospital and registered with the University Hospital Medical Network (UMIN)-clinical trials registry. The registration title is “UMIN000021434, Effect of exercise on structure and blood flow of choroid in normal subjects” (March 14, 2016).The participants were recruited between March 18, 2016 and March 31, 2016. Individuals working at the Sapporo City General Hospital were invited to the study by referral.

### Inclusion and exclusion criteria

**Fig 1:**
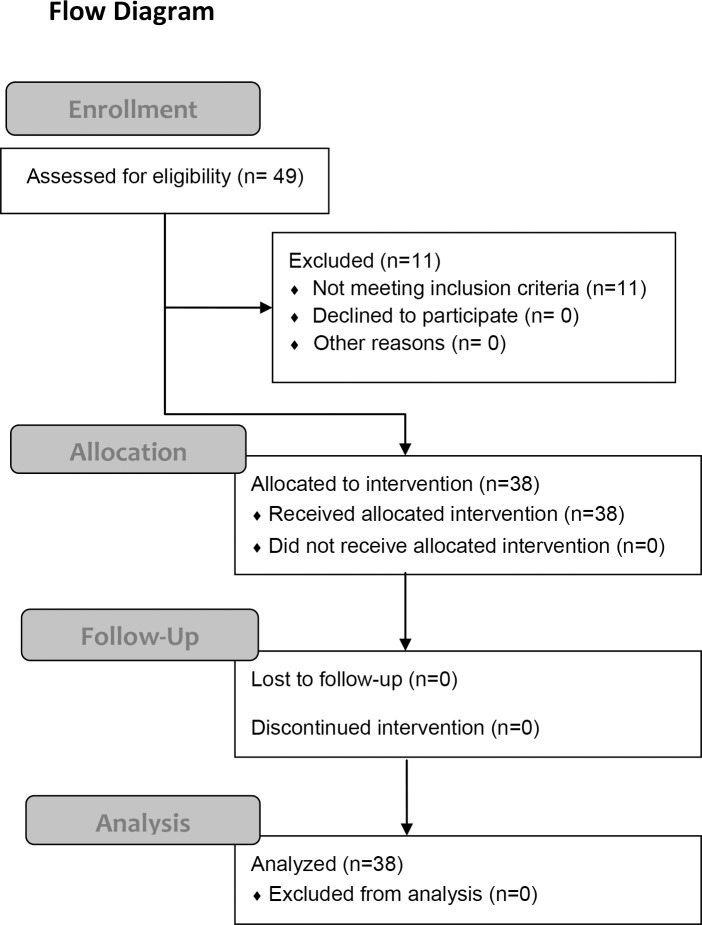
Flow diagram of all participants through the trial.

This was a prospective, cross sectional, interventional study of 49 right eyes of 49 normal, non-smoking individuals with no ophthalmic or systemic disorders. All individuals from which a written informed consent was obtained were assessed for eligibility. The exclusion criteria included an age of <18 years or>60 years, high myopia defined as a refractive error (spherical equivalent) greater than -6.0 diopters or an axial length of >26.5 mm, poor quality EDI-OCT image defined as an image index of <30, abnormal EDI-OCT findings, and previous ocular surgeries. Preliminary ophthalmologic examinations were performed to determine whether ocular abnormalities such as high myopia, corneal diseases, cataracts, vitreoretinal diseases, and glaucoma were present. Subjects with any systemic disorderssuch as hypertension and diabetes mellitus were also excluded based on the results of their most recent physical examination. The flow diagram displaying the progress of all participants through the trial is shown in Fig 1.

### Protocol of examinations

All examinations were performed and the data were collected at Sapporo City General Hospital by three investigators (TK, JM, and NO). On the day of the examination, participants were instructed not to take any alcohol and caffeine, and not to perform vigorous exercise. Otherwise, they were instructed to behave, drink, and eat according to their usual daily activities until an hour before the examinations. Then, they were instructed not to ingest anything from an hour before to the end of the examinations.

After a rest period of 20 minutes, the measurements of the systemic and ocular examinations at the baseline were performed [12,22]. All examinations were performed in the sitting position in the following sequence at each time point. Initially, the IOP (icare® TA01i, Icare Finland Oy, Finland) was measured which was followed by the EDI-OCT imaging with simultaneous measurements of the blood pressure, heart rate (HR), and peripheral oxygen saturation (SpO_2_), and lastly, the choroidal blood velocity was measured. Immediately after the completion of examination at the baseline, the exercise was started. All subjects underwent the Master’s single two-step exercise test. The number of steps was determined by age, sex, and weight of the subjects, and the number ranged from 114 to 162 steps [23]. The degree of the work load experienced by each participant was determined using the calculation of the percentage of the maximumheart-rate capacity (%HRmax) according to the following formula: %HRmax = 100 × (HR immediately after the exercise − HR at baseline)/(maximum HR–HR at baseline). The maximum HR was calculated as (220 –age in years) [7]. The same set of measurements was taken immediately after the exercise, and again 10 minutes after the end of the exercise. Oneset of measurement took approximately 2 minutes. In a previous study using Master's single two-step exercise, the heart rate was increased by ≥10 beats/min in 14% of the normal subjects at 5 min after the exercisecompared to that before exercise [24]. Another study using more intensive dynamic exercise reported that choroidal blood flow and choroidal blood velocity was stabilized within 10 minutes after the exercise [6]. We determined the timepoints after the exercise from these findings.

### Systemic and ophthalmic examinations

The systolic blood pressure (SBP), diastolic blood pressure (DBP), HR, SpO_2_, intraocular pressure (IOP), choroidal blood flow velocity, and EDI-OCT images were measured at each time point. The SBP, DBP, and HR were measured on the right arm with a commercial sphygmomanometer (HEM-759P, OMRON, Japan). The SpO_2_ was measured with a pulse oximeter (N560, Medtronic, USA) with the sensor placed on a finger of the left hand. The mean arterial pressure (MAP) and the mean ocular perfusion pressure (MOPP) were calculated according to the following formulas and used for the analyses: MAP = DBP + 1/3 (SBP—DBP), and MOPP = 2/3 MAP–IOP.

The central corneal thickness, the refractive error (spherical equivalent), and the axial length were also measured before the exercise.

### Measurement of choroidal blood flow velocity

**Fig 2:**
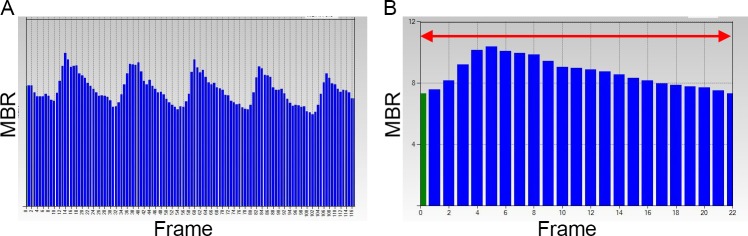
Measurement of avearge mean blur rate with LSFG. (A) Pulse waves recorded by LSFG showing the fluctuations in the MBR of the choroidal blood flow during each cardiac cycle for 4 seconds. The total number of frames is 118 in onescan. (B) Synthesized MBR image showing the average fluctuation in the MBR in one heartbeat. The red arrow indicates one heartbeat.

The choroidal blood flow velocity was determined by LSFG (LSFG-NAVI, Softcare, Fukuoka, Japan). The principles of LSFG have been described in detail [25–30]. Briefly, the instrument uses a diode laser with a wavelength of 830 nm to detect the movement of the red blood cells (RBCs) in the blood vessels. The light scattered from the targeted tissue creates speckle patterns on the image plane where the sensor is focused, and the moving RBCs produce a blur in the speckle patterns. The degree of blurring depends on the average velocity of the RBCs, and thus the mean blur rate (MBR), is a quantitative measure of the relative blood flow velocity. The MBR images are acquired at a rate of 30 frames/sec over 4 sec. Then, the MBR image during one heart beat is synthesized, and the mean value of the synthesized MBR image was determined as the average MBR which was used for analyses (Fig 2). Each image was recorded with the eye tracking system without pupil dilation.

To evaluate the average MBR of the choroid, the area of a rectangle (200 × 200 pixels, 5 × 5 degree) centered on the fovea was examined because this area is almost free of retinal vessels [30]. The central 5-degree square examined by LSFG is comparable to a square of approximate 1,500 μm length on the fundus of an emmetropic eye [31]. All LSFG examinations were performed by an experienced orthoptist under standardized mesopic lighting conditions. The average MBR was measured two times at each time point in all eyes, and the average of two measurements was used for the statistical analyses.

### Enhanced depth imaging optical coherence tomography (EDI-OCT)

EDI-OCT was performed with the Spectralis OCT instrument (Heidelberg Engineering, Heidelberg, Germany) at each time point. The methods used to obtain the EDI-OCT images were described in detail previously [16]. Horizontal cross sectional images of 30 degrees centered on the fovea were obtained for each eye, and each image was recorded usingthe eye tracking system without pupil dilation. Then, 100 scans were averaged to improve the signal-to-noise ratio. The same position of the fundus for each subject was scanned using the follow-up mode. The first scan at the baseline was set as the reference point for each subject, and subsequent scans after exercise were aligned to this. All OCT scans were performed by an experienced orthoptist under standardized mesopic lighting conditions to minimize the possible light-evoked vasodilations and constrictions.All images were obtained between 15:00 and 18:00 hours to minimize the effect of the diurnal variations in the choroidal structures [16, 32–34].

The retinal and choroidal areas of 1500 μm wide centered on the fovea were examined. The parameters measured includedthe central choroidal thickness (CCT), luminal, stromal, and total choroidal areas, the central foveal thickness (CFT), and retinal area. The L/C ratio was calculated. The CFT was defined as the distance fromthe internal limiting membrane to the outer surface of the retinal pigment epithelium (RPE), and the CCT as the distance from the outer border of the RPE to the chorioscleral interface. These distances were measured by two independent investigators (JM and KS) withthe caliper function of the software embedded in the OCT instrument. The averages of two measurements were used for the statistical analyses.

### Evaluation of luminal, stromal and total choroidal areas by binarization technique

The EDI-OCT images were evaluated by one of the authors (KS) who was masked to the clinical findings. The binarization of the choroidal area in the EDI-OCT images was performedby a modified Niblack method using the freely available software (ImageJ version 1.47, NIH, Bethesda, MD, USA) as described in detail [14, 16]. The choroidal area of 1500 μm wide centered on the fovea was examined. The area extended vertically from the RPE to the chorioscleral border. In the binarized images, the light pixels and the dark pixels were defined as the stromal and luminal areas, respectively. After addingthe data on the relationshipbetween the distance on the fundus and the pixels in the EDI-OCT images, the luminal and stromal areas were automatically calculated. Similarly, a retinal area of 1500 μm wide centered on the fovea was determined by measuring the area between internal limiting membrane and the outer border of the RPE.

All retinal and choroidal parameters were measured three times, and the averages of three measurements were used for the statistical analyses. Although the repeatability and reproducibility of the binarization method used for the analysis was presented to be high in normal eyes [14–17, 21], the intra-rater correlation coefficients were calculated for the data obtained from the images recorded at baseline.

The primary outcome measures are parameters obtained with OCT and LSFG before and after the exercise. The secondary outcome measures included the correlations between the OCT parameters and the hemodynamic parameters.

### Statistical analyses

Statistical analyses were performed with the SPSS version 22 software (IBM, Armonk, New York, USA). The significances of the changes in the systemic and ocular parameters before and after the exercise weredetermined by the repeated-measures analysis of variance with the Greenhouse-Geisser corrections. The Bonferroni test was used for post hoc analysis. The correlations among systemic parameters and choroidal parameters including the MBR and OCT parameters were determined by partial regression coefficients of correlation. The intra-rater correlation coefficients were calculated using 1-way random effects model for measurements of agreement. A two-sided *P* value of <0.05 was considered statistically significant.

## Results

### Baseline demographic data

Forty-nine subjects consisting of 20 men and 29 women were studied. Six eyes of 6 subjects were excluded because of high myopia, three eyes because of poor quality EDI-OCT images at baseline, one eye because of poor quality LSFG images at baseline, and one eye because of ocular hypertension. In the end, 38 subjects (19 men and 19 women) underwent the exercise, and the data from 38 eyes of 38 subjects were used for the statistical analyses (Fig 1). No adverse events were found during the study. The mean age of the subjects was 39.3 ± 9.6 years (± SD) with arange from 24 to 58 years. The mean axial length, the refractive errors, and the central corneal thickness were 24.6 ± 0.99 mm, -2.6 ± 1.75 diopters, and 536.4 ± 29.78 μm, respectively.

### Repeatability of measurements of OCT parameters

The intra-rater agreement was high, with an intraclass correlation coefficient of 0.999 (CI 0.999–1.000) for the total choroidal area, 0.997 (CI 0.994–0.998) for the luminal area, 0.976 (CI 0.959–0.987) for the stromal area, and 0.964 (CI 0.938–0.980) for the L/C ratio.

### Changes in the hemodynamic parameters

**Fig 3:**
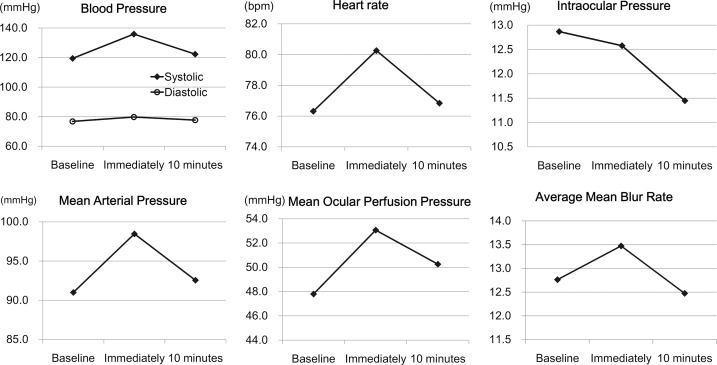
Changes in the hemodynamic parameters. All hemodynamicparameters were significantly increased immediately after the exercise, and they decreased at 10 minutes after the exercise.

The mean SBP, DBP, MAP, MOPP, and HR were significantly increased immediately after the exercise compared to that at the baseline (Table 1, Fig 3). The mean SBP and MOPP were significantly decreased at 10 minutes after the exercise compared to thoseimmediately after the exercise but were still significantly larger than that at the baseline. The mean MAP and HR at 10 minutes after exercise returned to the level at the baseline. The mean %HRmax was 4.0 ± 7.83%. There was a significant decrease in the IOP at 10 min after the exercise. There was no significant change in the SpO_2_ (*P* = 0.560) after the exercise.

**Table 1:** Changes in the hemodynamic parameters.

	Baseline	Immediately after exercise	*P*^1^	10 minutes after exercise	*P*^2^	*P*^3^
SBP	119.4 ± 14.64	135.9 ± 16.76 (+14.2 ± 10.37%)	<0.001	122.3 ± 14.49 (+2.6 ± 5.94%)	0.030	<0.001
DBP	76.0 ± 10.40	79.8 ± 11.74 (+5.3 ± 10.34%)	0.024	77.7 ± 10.82 (+2.2 ± 3.99%)	0.938	0.623
Heart rate	76.3 ± 12.36	80.3 ± 15.30 (+5.1 ± 9.33%)	0.006	76.8 ± 13.68 (+0.8 ± 9.02%)	1.000	0.063
IOP	12.9 ± 2.91	12.6 ± 2.60 (-1.1 ± 13.74%)	0.908	11.4 ± 2.45 (-10.3 ± 9.90%)	<0.001	<0.001
MAP	90.5 ± 10.71	98.5 ± 12.36 (+9.1 ± 9.12%)	<0.001	92.6 ± 11.04 (+2.6 ± 5.95%)	0.280	<0.001
MOPP	47.4 ± 7.68	53.1 ± 8.45 (+12.5 ± 12.40%)	<0.001	50.3 ± 8.16 (+6.3 ± 10.46%)	0.002	0.020
SpO_2_	98.3. ± 0.98	98.3 ± 0.96 (-0.1 ± 0.71%)	1.000	98.2 ± 1.01 (-0.13 ± 1.09%)	1.000	1.000
MBR	12.8 ± 4.27	13.5 ± 4.20 (+7.0 ± 13.38%)	0.006	12.5 ± 4.06 (-2.2 ± 12.70%)	0.672	<0.001

DBP, diastolic blood pressure; IOP, intraocular pressure; MAP, mean arterial pressure; MBR, mean blur rate; MOPP, mean ocular perfusion pressure; SBP, systolic blood pressure; SpO_2_, peripheral oxygen saturation.

^1^Significance between baseline and immediately after exercise

^2^Significance between baseline and 10 minutes after exercise

^3^Significance between immediately after exercise and 10 minutes after exercise

Changes from baseline are presented in parenthesis as relative to the baseline values.

**Fig 4:**
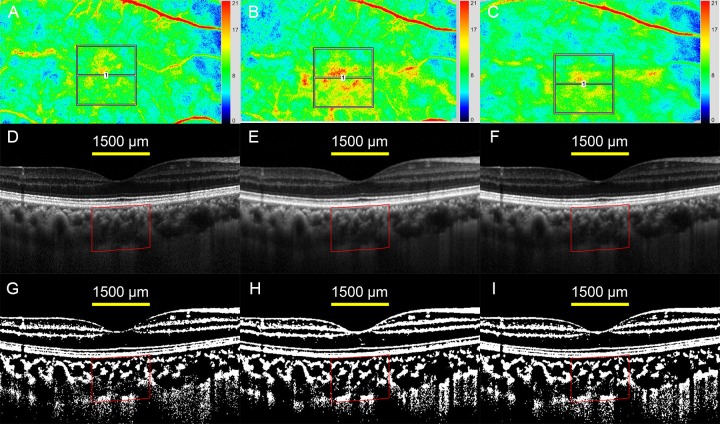
Two-dimensional color mappings of MBR in laser speckle flowgraphy (LSFG), enhanced depth imaging optical coherence tomographic (EDI-OCT) images, and converted binary images of a healthy 45-year-old woman. (A, B, C) LSFG images at baseline (A), immediately after the exercise (B), and 10 minutes after the exercise (C). A false-color composite map at the macula was created using the LSFG software. The red area indicates a faster blood flow, and the blue area indicates a slower blood flow. The average MBR was 9.3 (arbitrary unit) at the baseline which increased to 11.4 at immediately after the exercise, and then decreased to 9.7 at 10 minutes after the exercise. (D, E, F) EDI-OCT images at baseline (D), immediately after the exercise (E), and 10 minutes after the exercise (F). (G, H, I) The converted binary images of the EDI-OCT images shown in D (G), E (H), and F (I). The choroidal thickness and choroidal area did not change before and after the exercise.

The average MBR wassignificantly increased immediately after the exercise and it returned to the baseline levelat 10 minutes after the exercise (Table 1, Figs 3 and 4).

There were no significant differences in the changes in the hemodynamic parameters between young subjects (<40 years of age) and middle-aged (≥40 years of age) subjects and between sexes.

### Changes in optical coherence tomographic parameters

**Fig 5:**
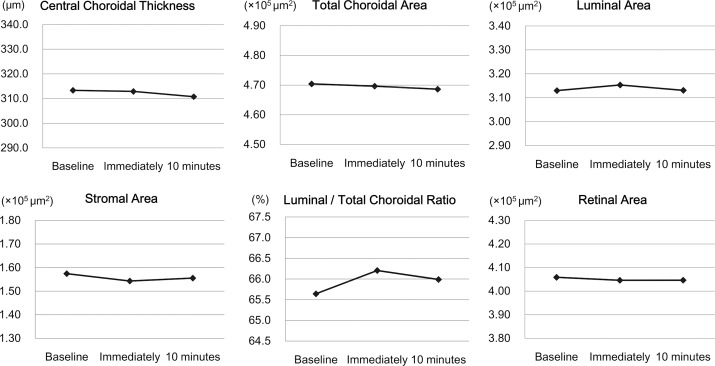
Changes in the optical coherence tomographic parameters. There were no significant changes in the mean choroidal and retinal parameters.

There were no significant changes in the mean choroidal parameters (Table 2, Fig 4, Fig 5). There were no significant differences in the changes in the OCT parameters between young and middle-aged subjects and between sexes.However, There were changes of >10 μm in the CCT in 12 (31.6%) subjects (S1 Table). For this reason, the changes after the exercise were examined relative to the baseline values which were defined as the values immediately after the exercise divided by the values at the baseline.

**Table 2:** Changes in the optical coherence tomographic parameters.

	Baseline	Immediately after exercise	10 minutes after exercise	*P*^1^
Central choroidal thickness (μm)	313.3 ± 91.96	312.9 ± 95.37 (-0.1 ± 2.51%)	310.8 ± 94.85 (-0.8 ± 2.39%)	0.065
Total choroidal area (x 10^5^ μm^2^)	4.70 ± 1.37	4.70 ± 1.43 (-0.2 ± 2.25%)	4.69 ± 1.41 (-0.4 ± 2.04%)	0.471
Luminal area (x 10^5^ μm^2^)	3.13 ± 1.09	3.15 ± 1.15 (+0.7 ± 3.52%)	3.13 ± 1.11 (+0.1 ± 3.70%)	0.587
Stromal area (x 10^5^ μm^2^)	1.58 ± 0.34	1.54 ± 0.35 (-2.0 ± 6.37%)	1.56 ± 0.38 (-1.4 ± 5.95%)	0.190
L/C ratio (%)	65.6 ± 4.00	66.1 ± 4.22 (+0.9 ± 2.59%)	66.0 ± 4.32 (+0.6 ± 2.81%)	0.182
Retinal area (x 10^5^ μm^2^)	4.06 ± 0.27	4.05 ± 0.27 (-0.3 ± 1.54%)	4.05 ± 0.28 (-0.3 ± 1.69%)	0.304
Central foveal thickness (μm)	231.9 ± 22.13	231.2 ± 22.35 (-0.4 ± 1.11%)	231.9 ± 22.47 (0.0 ± 0.93%)	0.096

^1^repeated-measures analysis of variance with Greenhouse-Geisser corrections

Changes from baseline are presented in parenthesis as relative to the baseline values.

### Correlations among OCT parameters, systemic parameters, and choroidal blood velocity

The changes in the CCT were significantly correlated with thoseof the total choroidal area (r = 0.852, *P*<0.001) and the luminal area (r = 0.653, *P*<0.001) in partial regression coefficient in which age and the axial length were set as the control variables. But there was no significant correlation between the changes in the CCT and thoseof the stromal area (r = 0.139, *P* = 0.420). The changes in the CCT were significantly correlated with those of DBP, MAP, MOPP, and MBR (Table 3). The changes in the total choroidal area were significantly correlated with those of the DBP, MAP, and MOPP, but the changes in the luminal area were not significantly correlated with thoseof the systemic parameters. The change of MBR was significantly correlated with that of the systemic parameters.

**Table 3:** Correlations of changing rate of OCT parameters with those of systemic parameters and MBR.

	SBP	DBP	MAP	IOP	MOPP	MBR
Central choroidal thickness	r = 0.310 *P* = 0.066	r = 0.439 *P* = 0.007	r = 0.458 *P* = 0.005	r = 0.150 *P* = 0.383	r = 0.393 *P* = 0.018	r = 0.376 *P* = 0.024
Total choroidal area	r = 0.222 *P* = 0.193	r = 0.455 *P* = 0.005	r = 0.428 *P* = 0.009	r = 0.203 *P* = 0.234	r = 0.347 *P* = 0.038	r = 0.174 *P* = 0.311
Luminal area	r = 0.063 *P* = 0.715	r = 0.254 *P* = 0.136	r = 0.207 *P* = 0.226	r = 0.076 *P* = 0.658	r = 0.197 *P* = 0.249	r = 0.246 *P* = 0.149
Stromal area	r = 0.235 *P* = 0.167	r = 0.213 *P* = 0.212	r = 0.263 *P* = 0.122	r = 0.111 *P* = 0.520	r = 0.200 *P* = 0.243	r = -0.095 *P* = 0.580
L/C ratio	r = -0.155 *P* = 0.505	r = -0.061 *P* = 0.725	r = -0.101 *P* = 0.557	r = -0.106 *P* = 0.539	r = -0.025 *P* = 0.884	r = 0.176 *P* = 0.304

L/C ratio, ratio of luminal area to total choroidal area; SBP, systolic blood pressure; DBP, diastolic blood pressure; IOP, intraocular pressure; MAP, mean arterial pressure; MOPP, mean ocular perfusion pressure.

## Discussion

Our results showed that there were significant changes in all of the systemic parameters except for the SpO_2_ after the exercise. The values of the parameters were significantly increased immediately after the exercise and decreased significantly at 10 minutes after the exercise. The mean MBR was also increased significantly immediately after the exercise and returned to the baseline level at 10 minutes after the exercise, which agrees with earlier findings [6,35]. These results suggest that a restperiod is needed before the measurements ofthe choroidal hemodynamics in normal subjects. A rest period should also be used before examining the choroidal hemodynamics in diseased eyes such as glaucoma and ischemic optic neuropathy in which the choroidal circulation and regulation of ocular blood flow have been reported to be altered [36,37]

There were no significant changes in the mean CCT before and after the exercise which is consistent with recent reports [2, 13]. Our results added the new information that there were no significant changes in the mean total choroidal area, the mean luminal and stromal areas after the dynamic exercise.

Exercise causes an increase in the MOPP and blood velocity in the choroidal vessels. Indeed, the mean MOPP and the mean MBR were increased significantly immediately after the exercise compared to that at baseline. If the choroidal vessels are completely regulated, the luminal area should decrease because the choroidal arterioles constrict to keep the choroidal blood flow constant. This may be accompanied by a decrease in the total choroidal area and CCT. Conversely, if the choroidal vessels are not regulated, the luminal area shouldincrease due to a passive expansion by the increase in the MOPP. This may be accompanied by an increase in the total choroidal area and CCT. In this study, the mean luminal area, the mean total choroidal area, and the mean CCT were not significantly changed, which suggests that the choroidal vessels are partially regulated. It has been reported that the choroidal blood flow is regulated during dynamic and isometric exercise in spite of the increase in the choroidal blood velocity [6,9,11,22]. Our results are compatible with those findings although we did not examine the choroidal blood flow itself.

Although there were no significant changes in the mean values of the choroidal parameters, the change in the CCT was significantly correlated with those of the total choroidal area and luminal area but not with that of the stromal area. Thesefindings suggest that the changes in the CCT during the exercise were most likely due to the changes in the luminal area. However, the changes of the luminal area were not significantly correlated with the circulatory parameters, which suggests that the changes in the CCT and total choroidal area may be influenced by other factors than that examined in this study.

It is known that the choroidal vessels are under neurogenic control [38–40]. Exercise activates the sympathetic nervous system [41]which causes a constriction of the choroidal vessels. In addition, the vascular tone and diameters can be altered by various factors including blood gases and pH, visual stimulation, and vasoactive agents including endothelin-I and nitric oxide [5, 9–11]. We considered that some of these may have influenced the choroidal luminal size during the exercise.

The CCT and total choroidal area were significantly correlated with the DBP but not with the SBP. Although the reason for this is unclear, it is known that the reactivity to physical activity is different for the SBP and DBP [35,42,43]. The SBP has been reported to increase during exercise consistently in different studies due to the increase in the cardiac output and vasoconstriction in the non-exercising vascular bed. In contrast, the DBP can be decreased or increased. In this study, the SBP was increased in almost all subjects immediately after the exercise, but the DBP was increased in 63.2% of subjects and decreased in 34.2% (S2 Table).The decrease in the DBP may be caused by a vasodilation of the muscle vasculaturewhich would result in a decrease in the venous return to the heart and blood volume circulating the extramuscular organs and tissues including the choroid [35,43]. Conversely, an increase in the DBP couldbe caused by the occlusion of the muscle vasculature due to the forceful contractions of the exercising muscle during intense exercise, which would result in an increase in the venous return and blood volume circulating the choroid. We suggest that these mechanisms may be related to the positive correlations between the changes in the DBP and those in the CCT and the total choroidal area.

Changes in the IOP could also influence the choroidal parameters. Many studies have reported that the IOPs were decreased after exercise [1–5, 7, 8]. Hong et al reported that IOPs were significantly decreased after exercise with no correlation with the choroidal thickness [2], which is consistent with our results. The changes in the IOP were not correlated with those of the choroidal structural parameters, suggesting that IOP may be less likely related to the choroidal structural changes after the exercise.

Because the hemodynamic changes are similar between the dynamic and isometric exercises, the changes in the choroidal structure might be similar between the two types of exercises. However, this may not be true for changes during the exercise with Valsalva maneuver mechanisms in which the blood pressure and IOP change markedly. Recent studies reported contradictory results on the choroidal thickness during Valsalva maneuver [44,45]. The intensity and duration of exercise may also influence the results. Further studies are needed to determine the effect of different exercise routines on the IOP and choroidal vascular system.

There are some limitations in this study. First, the measurements of choroidal thickness and total choroidal area using the manual delineation of chorioscleral borders are not completely objective although we excluded the 3 subjects with poor EDI-OCT image quality. Second, the sample size was small. Third, the range of the age was large. The detection of the physiological phenomenon may be easier in younger subjects than in middle-aged subjects. Ethnic differences in the choroidal regulatory mechanisms might also exist. Lastly, the protocol of Master’s single two-step method is partially rather than totally quantified although the degree of the work load was determined by the protocol of the method. Consideration of the %HR max may be more rigorous for the determination of the work load on the basis of similar work load. The %HRmax (mean ± SD) in this study was 4.0 ± 7.83% which was lower than that reported by previous studies [7]. However, to the best of our knowledge, this is the first report that examined the changes in the luminal and stromal area of choroid after exercise.

In conclusion, there were no significant changes in the mean CCT and the mean choroidal area after the exercise although there were significant changes in the systemic hemodynamic parameters and choroidal blood flow velocity. These findings indicate that mild dynamic exercise is unlikely to alter the choroidal thickness and area.

## Supporting Information

S1 TREND Checklist TREND Checklist. (PDF)

S1 Table Choroidal parameters of all subjects. (DOCX)

S2 Table Hemodynamic parameters of all subjects. (DOCX)
