# Supplementary material for: Effects of Exercise on the Structure and Circulation of Choroid in Normal Eyes
Source: PLoS One. 2016 Dec 14;11(12):e0168336. doi: 10.1371/journal.pone.0168336 (PMC5156418; doi:10.1371/journal.pone.0168336)
Supplement: S1 Table — (DOCX) [file pone.0168336.s002.docx]

**S1 Table. Choroidal parameters of all subjects**

| case | Age | Sex | MBRb | MBRim | MBR10 | CCTb | CCTim | CCT10 | TCAb | TCAim | TCA10 | LAb | LAim | LA10 | SAb | SAim | SA10 |
| --- | --- | --- | --- | --- | --- | --- | --- | --- | --- | --- | --- | --- | --- | --- | --- | --- | --- |
| 01 | 46 | M | 22.1 | 23.2 | 22.4 | 392.2 | 398.6 | 403.8 | 5.80 | 5.97 | 5.98 | 4.28 | 4.12 | 4.41 | 1.51 | 1.85 | 1.56 |
| 02 | 46 | M | 8.5 | 9.9 | 9.7 | 242.5 | 234.8 | 239.9 | 3.43 | 3.36 | 3.43 | 2.19 | 2.17 | 2.22 | 1.24 | 1.19 | 1.21 |
| 03 | 30 | M | 13.2 | 14.8 | 12.3 | 291.5 | 294.1 | 290.3 | 4.39 | 4.45 | 4.39 | 2.74 | 2.78 | 2.81 | 1.65 | 1.67 | 1.59 |
| 04 | 52 | F | 7.7 | 8.1 | 8.0 | 187.1 | 179.3 | 180.6 | 2.84 | 2.77 | 2.82 | 1.70 | 1.67 | 1.70 | 1.14 | 1.10 | 1.12 |
| 05 | 49 | M | 15.0 | 15.4 | 14.4 | 494.1 | 501.8 | 497.9 | 7.36 | 7.43 | 7.44 | 4.96 | 4.89 | 4.67 | 2.40 | 2.55 | 2.77 |
| 06 | 24 | F | 13.7 | 15.8 | 13.0 | 470.9 | 469.6 | 479.9 | 6.99 | 6.95 | 7.15 | 5.14 | 5.18 | 5.10 | 1.85 | 1.77 | 2.05 |
| 07 | 58 | M | 16.1 | 15.7 | 15.2 | 203.8 | 198.7 | 206.4 | 3.09 | 3.06 | 3.12 | 1.84 | 1.79 | 1.87 | 1.26 | 1.28 | 1.24 |
| 08 | 30 | F | 11.3 | 13.3 | 10.7 | 289.0 | 288.7 | 287.7 | 4.33 | 4.30 | 4.34 | 2.79 | 2.88 | 2.82 | 1.53 | 1.42 | 1.52 |
| 09 | 38 | F | 11.8 | 12.2 | 14.7 | 258.0 | 255.4 | 249.0 | 3.83 | 3.77 | 3.73 | 2.39 | 2.39 | 2.38 | 1.44 | 1.38 | 1.35 |
| 10 | 33 | M | 9.7 | 8.3 | 7.4 | 350.9 | 338.0 | 340.6 | 5.31 | 5.15 | 5.19 | 3.80 | 3.54 | 3.58 | 1.51 | 1.61 | 1.61 |
| 11 | 44 | F | 9.6 | 13.7 | 10.9 | 255.4 | 261.9 | 249.0 | 4.04 | 3.99 | 3.96 | 2.58 | 2.60 | 2.61 | 1.45 | 1.38 | 1.35 |
| 12 | 44 | F | 11.8 | 13.4 | 12.4 | 255.4 | 260.6 | 256.7 | 3.93 | 3.94 | 3.90 | 2.50 | 2.47 | 2.39 | 1.43 | 1.47 | 1.50 |
| 13 | 32 | M | 9.0 | 9.9 | 8.0 | 325.1 | 316.1 | 322.5 | 4.99 | 4.80 | 5.04 | 3.34 | 3.11 | 3.53 | 1.65 | 1.69 | 1.51 |
| 14 | 40 | F | 6.1 | 9.1 | 7.9 | 247.7 | 255.4 | 250.3 | 3.82 | 3.93 | 3.84 | 2.53 | 2.67 | 2.54 | 1.29 | 1.25 | 1.29 |
| 15 | 47 | F | 10.7 | 10.5 | 8.9 | 153.5 | 141.9 | 148.4 | 2.37 | 2.24 | 2.38 | 1.37 | 1.35 | 1.38 | 1.00 | 0.89 | 1.00 |
| 16 | 47 | F | 11.9 | 12.4 | 12.8 | 343.1 | 352.2 | 348.3 | 5.30 | 5.38 | 5.32 | 3.43 | 3.57 | 3.60 | 1.87 | 1.81 | 1.72 |
| 17 | 59 | M | 13.1 | 11.1 | 11.5 | 354.8 | 353.5 | 352.2 | 5.28 | 5.37 | 5.34 | 3.49 | 3.65 | 3.59 | 1.79 | 1.72 | 1.76 |
| 18 | 55 | F | 10.9 | 11.4 | 10.3 | 247.7 | 246.4 | 246.4 | 3.69 | 3.65 | 3.66 | 2.31 | 2.30 | 2.40 | 1.38 | 1.35 | 1.26 |
| 19 | 46 | F | 10.1 | 10.7 | 8.9 | 263.2 | 269.6 | 267.0 | 3.98 | 4.07 | 3.97 | 2.59 | 2.66 | 2.56 | 1.39 | 1.41 | 1.42 |
| 20 | 38 | F | 9.9 | 11.2 | 10.6 | 281.2 | 279.9 | 277.4 | 4.16 | 4.19 | 4.18 | 2.64 | 2.72 | 2.71 | 1.52 | 1.46 | 1.48 |
| 21 | 43 | F | 15.3 | 17.0 | 15.1 | 212.9 | 215.4 | 207.7 | 3.21 | 3.24 | 3.18 | 2.06 | 2.13 | 2.07 | 1.16 | 1.11 | 1.11 |
| 22 | 38 | F | 14.3 | 14.5 | 13.2 | 298.0 | 296.7 | 298.0 | 4.43 | 4.38 | 4.43 | 2.87 | 2.83 | 2.87 | 1.56 | 1.56 | 1.56 |
| 23 | 46 | M | 8.2 | 7.6 | 8.0 | 380.6 | 381.8 | 381.8 | 5.77 | 5.75 | 5.80 | 3.80 | 3.84 | 3.83 | 1.97 | 1.91 | 1.98 |
| 24 | 24 | F | 13.1 | 13.2 | 11.8 | 256.7 | 242.5 | 238.7 | 3.81 | 3.60 | 3.60 | 2.31 | 2.24 | 2.11 | 1.51 | 1.36 | 1.48 |
| 25 | 33 | M | 17.6 | 18.0 | 17.8 | 473.4 | 464.4 | 455.4 | 7.15 | 6.98 | 6.88 | 4.86 | 4.80 | 4.49 | 2.30 | 2.19 | 2.39 |
| 26 | 28 | M | 12.6 | 11.8 | 11.2 | 406.4 | 402.5 | 398.6 | 6.14 | 6.11 | 5.98 | 3.94 | 3.87 | 3.77 | 2.20 | 2.24 | 2.21 |
| 27 | 23 | M | 23.2 | 23.4 | 20.9 | 303.2 | 300.6 | 299.3 | 4.41 | 4.45 | 4.40 | 2.98 | 2.99 | 2.92 | 1.42 | 1.46 | 1.48 |
| 28 | 30 | M | 14.5 | 13.7 | 12.5 | 378.0 | 378.0 | 374.1 | 5.73 | 5.76 | 5.69 | 3.67 | 3.69 | 3.60 | 2.05 | 2.07 | 2.09 |
| 29 | 31 | F | 7.7 | 8.4 | 8.2 | 448.9 | 461.8 | 459.2 | 6.76 | 6.90 | 6.84 | 4.87 | 5.29 | 5.14 | 1.89 | 1.62 | 1.70 |
| 30 | 32 | M | 17.5 | 19.5 | 17.0 | 294.1 | 287.7 | 279.9 | 4.43 | 4.35 | 4.24 | 2.80 | 2.73 | 2.71 | 1.63 | 1.62 | 1.53 |
| 31 | 43 | M | 24.2 | 23.3 | 22.9 | 241.2 | 238.7 | 225.8 | 3.64 | 3.57 | 3.49 | 2.30 | 2.33 | 2.21 | 1.34 | 1.24 | 1.29 |
| 32 | 36 | M | 13.8 | 14.2 | 13.1 | 336.7 | 327.7 | 323.8 | 5.04 | 4.95 | 4.87 | 3.37 | 3.28 | 3.44 | 1.67 | 1.66 | 1.43 |
| 33 | 29 | M | 11.4 | 15.1 | 15.6 | 317.3 | 316.1 | 314.8 | 4.56 | 4.56 | 4.55 | 3.01 | 3.02 | 3.00 | 1.55 | 1.55 | 1.55 |
| 34 | 37 | F | 10.7 | 10.9 | 11.1 | 254.1 | 249.0 | 246.4 | 3.78 | 3.67 | 3.69 | 2.49 | 2.49 | 2.52 | 1.29 | 1.18 | 1.17 |
| 35 | 39 | M | 10.9 | 10.0 | 8.5 | 198.7 | 201.2 | 201.2 | 2.89 | 2.97 | 2.99 | 1.87 | 1.95 | 1.99 | 1.02 | 1.02 | 0.99 |
| 36 | 45 | F | 9.3 | 11.4 | 9.7 | 361.2 | 362.5 | 359.9 | 5.68 | 5.74 | 5.69 | 4.12 | 4.16 | 4.12 | 1.56 | 1.57 | 1.57 |
| 37 | 27 | M | 19.2 | 19.9 | 18.5 | 562.4 | 587.0 | 576.6 | 8.43 | 8.77 | 8.62 | 6.33 | 6.96 | 6.75 | 2.10 | 1.80 | 1.87 |
| 38 | 51 | F | 9.2 | 10.0 | 9.2 | 276.1 | 279.9 | 273.5 | 4.20 | 4.13 | 4.12 | 2.65 | 2.65 | 2.62 | 1.55 | 1.48 | 1.50 |

M; male, F; female, MBR; mean blur rate, CCT; central choroidal thickness, TCA; total choroidal area, LA; luminal area, SA; stromal area, b; at baseline, im; immediately after the exercise, 10; 10 minutes after the exercise

MBR has arbitrary units. CCT are expressed as μm. Choroidal areas are expressed as 10^5^ μm^2^.
