# Supplementary material for: Effects of Exercise on the Structure and Circulation of Choroid in Normal Eyes
Source: PLoS One. 2016 Dec 14;11(12):e0168336. doi: 10.1371/journal.pone.0168336 (PMC5156418; doi:10.1371/journal.pone.0168336)
Supplement: S2 Table — (DOCX) [file pone.0168336.s003.docx]

**S2 Table. Hemodynamic parameters of all subjects**

| case | SBPb | SBPim | SBP10 | DBPb | DBPim | DBP10 | MAPb | MAPim | MAP10 | IOPb | IOPim | IOP10 | MOPPb | MOPPim | MOPP10 | HRb | HRim | HR10 |
| --- | --- | --- | --- | --- | --- | --- | --- | --- | --- | --- | --- | --- | --- | --- | --- | --- | --- | --- |
| 01 | 94 | 130 | 116 | 76 | 101 | 93 | 82.0 | 110.7 | 100.7 | 14.0 | 14.0 | 13 | 40.7 | 59.8 | 54.1 | 82 | 119 | 101 |
| 02 | 132 | 143 | 140 | 88 | 94 | 89 | 102.7 | 110.3 | 106.0 | 11.0 | 13.0 | 11 | 57.4 | 60.6 | 59.7 | 71 | 71 | 73 |
| 03 | 123 | 152 | 128 | 72 | 81 | 77 | 89.0 | 104.7 | 94.0 | 15.0 | 15.0 | 14 | 44.3 | 54.8 | 48.7 | 90 | 103 | 77 |
| 04 | 122 | 114 | 126 | 62 | 60 | 96 | 82.0 | 78.0 | 106.0 | 9.0 | 7.0 | 8 | 45.7 | 45.0 | 62.7 | 81 | 78 | 108 |
| 05 | 136 | 138 | 124 | 86 | 71 | 85 | 102.7 | 93.3 | 98.0 | 16.0 | 14.0 | 10 | 52.4 | 48.2 | 55.3 | 105 | 109 | 96 |
| 06 | 105 | 106 | 107 | 66 | 65 | 69 | 79.0 | 78.7 | 81.7 | 10.0 | 10.0 | 9 | 42.7 | 42.4 | 45.4 | 80 | 79 | 79 |
| 07 | 128 | 150 | 136 | 79 | 76 | 84 | 95.3 | 100.7 | 101.3 | 14.0 | 13.0 | 12 | 49.6 | 54.1 | 55.6 | 91 | 103 | 98 |
| 08 | 101 | 128 | 96 | 58 | 65 | 62 | 72.3 | 86.0 | 73.3 | 16.0 | 12.0 | 15 | 32.2 | 45.3 | 33.9 | 72 | 85 | 71 |
| 09 | 114 | 145 | 115 | 76 | 85 | 78 | 88.7 | 105.0 | 90.3 | 19.0 | 18.0 | 16 | 40.1 | 52.0 | 44.2 | 74 | 83 | 82 |
| 10 | 128 | 128 | 128 | 77 | 75 | 75 | 94.0 | 92.7 | 92.7 | 11.0 | 14.0 | 11 | 51.7 | 47.8 | 50.8 | 89 | 91 | 90 |
| 11 | 117 | 142 | 112 | 75 | 87 | 77 | 89.0 | 105.3 | 88.7 | 14.0 | 13.0 | 13 | 45.3 | 57.2 | 46.1 | 69 | 72 | 70 |
| 12 | 98 | 125 | 98 | 65 | 71 | 71 | 76.0 | 89.0 | 80.0 | 10.0 | 13.0 | 10 | 40.7 | 46.3 | 43.3 | 65 | 67 | 66 |
| 13 | 123 | 152 | 132 | 83 | 97 | 89 | 96.3 | 115.3 | 103.3 | 13.0 | 14.0 | 13 | 51.2 | 62.9 | 55.9 | 96 | 99 | 93 |
| 14 | 100 | 125 | 99 | 70 | 83 | 70 | 80.0 | 97.0 | 79.7 | 11.0 | 12.0 | 12 | 42.3 | 52.7 | 41.1 | 94 | 100 | 93 |
| 15 | 114 | 124 | 118 | 78 | 77 | 69 | 90.0 | 92.7 | 85.3 | 17.0 | 17.0 | 16 | 43.0 | 44.8 | 40.9 | 68 | 64 | 62 |
| 16 | 94 | 96 | 107 | 59 | 68 | 54 | 70.7 | 77.3 | 71.7 | 11.0 | 14.0 | 10 | 36.1 | 37.6 | 37.8 | 59 | 57 | 60 |
| 17 | 135 | 137 | 138 | 87 | 90 | 90 | 103.0 | 105.7 | 106.0 | 10.0 | 12.0 | 10 | 58.7 | 58.4 | 60.7 | 75 | 74 | 78 |
| 18 | 114 | 119 | 118 | 68 | 67 | 68 | 83.3 | 84.3 | 84.7 | 14.0 | 13.0 | 15 | 41.6 | 43.2 | 41.4 | 61 | 63 | 56 |
| 19 | 103 | 116 | 104 | 68 | 68 | 64 | 79.7 | 84.0 | 77.3 | 11.0 | 9.0 | 9 | 42.1 | 47.0 | 42.6 | 68 | 70 | 69 |
| 20 | 122 | 146 | 125 | 78 | 86 | 80 | 92.7 | 106.0 | 95.0 | 19.0 | 17.0 | 16 | 42.8 | 53.7 | 47.3 | 68 | 71 | 67 |
| 21 | 111 | 138 | 116 | 72 | 76 | 74 | 85.0 | 96.7 | 88.0 | 11.0 | 11.0 | 11 | 45.7 | 53.4 | 47.7 | 77 | 84 | 76 |
| 22 | 120 | 151 | 120 | 70 | 69 | 77 | 86.7 | 96.3 | 91.3 | 11.0 | 10.0 | 9 | 46.8 | 54.2 | 51.9 | 60 | 68 | 69 |

| 23 | 140 | 160 | 139 | 87 | 98 | 75 | 104.7 | 118.7 | 96.3 | 9.0 | 9.0 | 7 | 60.8 | 70.1 | 57.2 | 85 | 83 | 73 |
| --- | --- | --- | --- | --- | --- | --- | --- | --- | --- | --- | --- | --- | --- | --- | --- | --- | --- | --- |
| 24 | 115 | 127 | 108 | 97 | 74 | 64 | 103.0 | 91.7 | 78.7 | 18.0 | 14.0 | 14 | 50.7 | 47.1 | 38.4 | 73 | 76 | 81 |
| 25 | 116 | 128 | 121 | 66 | 76 | 70 | 82.7 | 93.3 | 87.0 | 12.0 | 13.0 | 10 | 43.1 | 49.2 | 48.0 | 84 | 81 | 84 |
| 26 | 134 | 147 | 138 | 81 | 87 | 78 | 98.7 | 107.0 | 98.0 | 14.0 | 13.0 | 11 | 51.8 | 58.3 | 54.3 | 89 | 94 | 92 |
| 27 | 115 | 138 | 124 | 71 | 80 | 73 | 85.7 | 99.3 | 90.0 | 14.0 | 13.0 | 12 | 43.1 | 53.2 | 48.0 | 95 | 98 | 92 |
| 28 | 123 | 137 | 132 | 78 | 73 | 77 | 93.0 | 94.3 | 95.3 | 15.0 | 16.0 | 13 | 47.0 | 46.9 | 50.6 | 67 | 62 | 64 |
| 29 | 112 | 125 | 109 | 75 | 83 | 77 | 87.3 | 97.0 | 87.7 | 12.0 | 13.0 | 11 | 46.2 | 51.7 | 47.4 | 85 | 90 | 84 |
| 30 | 125 | 149 | 130 | 88 | 99 | 86 | 100.3 | 115.7 | 100.7 | 10.0 | 9.0 | 10 | 56.9 | 68.1 | 57.1 | 77 | 80 | 72 |
| 31 | 144 | 172 | 152 | 106 | 103 | 98 | 118.7 | 126.0 | 116.0 | 12.0 | 12.0 | 10 | 67.1 | 72.0 | 67.3 | 86 | 92 | 85 |
| 32 | 154 | 168 | 159 | 92 | 100 | 100 | 112.7 | 122.7 | 119.7 | 9.0 | 10 | 9 | 66.1 | 71.8 | 70.8 | 70 | 78 | 68 |
| 33 | 117 | 124 | 114 | 69 | 77 | 95 | 85.0 | 92.7 | 101.3 | 16 | 14 | 14 | 40.7 | 47.8 | 53.6 | 62 | 66 | 65 |
| 34 | 117 | 122 | 117 | 65 | 66 | 64 | 82.3 | 84.7 | 81.7 | 13 | 10 | 11 | 41.9 | 46.4 | 43.4 | 66 | 77 | 64 |
| 35 | 141 | 143 | 133 | 67 | 66 | 71 | 91.7 | 91.7 | 91.7 | 16 | 16 | 14 | 45.1 | 45.1 | 47.1 | 46 | 49 | 45 |
| 36 | 112 | 148 | 125 | 80 | 83 | 79 | 90.7 | 104.7 | 94.3 | 8 | 8 | 8 | 52.4 | 61.8 | 54.9 | 79 | 73 | 82 |
| 37 | 140 | 157 | 136 | 80 | 84 | 84 | 100.0 | 108.3 | 101.3 | 14 | 14 | 10 | 52.7 | 58.2 | 57.6 | 71 | 75 | 69 |
| 38 | 99 | 113 | 107 | 73 | 70 | 70 | 81.7 | 84.3 | 82.3 | 10 | 9 | 8 | 44.4 | 47.2 | 46.9 | 70 | 66 | 66 |

SBP; systolic blood pressure, DBP; diastolic blood pressure, MAP; mean arterial pressure, IOP; intraocular pressure, MOPP; mean ocular perfusion pressure, HR; heart rate, b; at baseline, im; immediately after the exercise, 10; 10 minutes after the exercise
